# A Generalizable and Interpretable Framework for Molecular Subtype Classification of Pancreatic Ductal Adenocarcinoma Integrating Conformal Uncertainty Quantification and Consensus-Based Explainable Artificial Intelligence Across Multiple Cohorts

**DOI:** 10.3390/ijms27156989

**Published:** 2026-08-04

**Authors:** Seyma Yasar, Fatma Hilal Yagin, Sarah A. Alzakari, Amal K. Alkhalifa, Fahaid Al-Hashem, Abedelmalek Kalefh Tabnjh

**Affiliations:** 1 Department of Biostatistics and Medical Informatics, Faculty of Medicine, İnönü University, Malatya 44280, Türkiye; 2 Department of Biostatistics, Faculty of Medicine, Malatya Turgut Ozal University, Malatya 44210, Türkiye; 3 Department of Computer Sciences, College of Computer and Information Sciences, Princess Nourah bint Abdulrahman University, P.O. Box 84428, Riyadh 11671, Saudi Arabia; 4 Department of Physiology, College of Medicine, King Khalid University, Abha 61421, Saudi Arabia; 5 Department of Cariology, Institute of Odontology, The Sahlgrenska Academy, University of Gothenburg, 40530 Gothenburg, Sweden; 6 Department of Applied Dental Sciences, Faculty of Applied Medical Sciences, Jordan University of Science and Technology, Irbid 22110, Jordan; 7 Department of Pathology, Saveetha Medical College and Hospital, Saveetha Institute of Medical and Technical Sciences, Chennai 602105, Tamil Nadu, India

**Keywords:** pancreatic ductal adenocarcinoma, molecular subtype, cross-cohort validation, conformal prediction, explainable AI, CPTAC, TCGA, TRIPOD+AI

## Abstract

Pancreatic ductal adenocarcinoma (PDAC) has two principal molecular subtypes—classical and basal-like—with divergent prognosis and chemotherapy response, yet transcriptomic classifiers rarely generalize across cohorts or quantify per-patient uncertainty. We trained a classical-versus-basal-like classifier on CPTAC-PDAC (*n* = 140) and externally validated it on histology-filtered TCGA-PAAD (*n* = 150). Twelve algorithms were benchmarked under stratified nested cross-validation with four-method consensus feature selection; domain adaptation (naive transfer, CORAL, and ComBat), four conformal procedures (split, weighted, CV+, and Conformal Risk Control), and a four-method consensus explainable-AI framework (SHAP, LIME, permutation importance, and decision-curve ablation) with pathway enrichment were then evaluated. Top models reached a cross-validated AUROC ≈ 0.96 and external AUROC 0.913–0.938 (top-3 ensemble 0.961); batch correction did not improve transfer, indicating minimal residual batch effect. Consensus explainability recovered keratinization biology and nominated five candidate genes (GSDMC, A2ML1, PIP5K1B, IL20RB, and AKR7L) beyond the Moffitt signature. All four conformal procedures plateaued near 0.85 coverage at α = 0.05 under zero-shot transfer, whereas local recalibration on a small target sample restored nominal coverage. We present a transparent, externally validated, uncertainty-aware and TRIPOD+AI-compliant PDAC subtype classifier, best deployed as a calibrated decision-support tool with site-specific recalibration.

## 1. Introduction

Pancreatic ductal adenocarcinoma (PDAC) remains one of the most lethal solid malignancies, with a five-year survival rate below 13% and an incidence that is projected to surpass breast cancer mortality within this decade [1,2]. Transcriptomic profiling over the past decade has consistently identified two dominant molecular subtypes—classical (pancreatic-progenitor-like) and basal-like (squamous, epithelial–mesenchymal–transition-enriched)—with divergent prognoses and differential responses to FOLFIRINOX (folinic acid, fluorouracil, irinotecan, and oxaliplatin) versus gemcitabine-based chemotherapy regimens [3,4,5]. Moffitt and colleagues defined a 50-gene signature that captures this dichotomy across bulk-tumor RNA-seq data [6], and the subsequent Purity Independent Subtyping of Tumors (PurIST) 16-gene single-sample classifier [7] further refined the framework into a clinically deployable assay. A recent synthesis nevertheless cautions that, despite this compelling biological rationale, routine clinical adoption of the classical/basal-like taxonomy remains constrained by limited cross-study reproducibility and practical implementation barriers, with GATA6 immunohistochemistry proposed as a pragmatic prognostic surrogate [8].

Clinically, PDAC is an aggressive ductal-epithelial malignancy marked by dense desmoplastic stroma, early perineural and lymphovascular spread, and a strong propensity for lymph-node metastasis, which remains its single most important prognostic determinant; even uncommon morphological variants such as plasmacytoid/poorly-cohesive forms and the extent of lymphatic-vessel invasion carry independent prognostic weight [9]. Fewer than one-fifth of tumors are resectable at diagnosis, and first-line systemic therapy is still selected largely empirically. Against this pathological backdrop, molecular subtyping is clinically meaningful because the basal-like/squamous program—enriched for epithelial–mesenchymal transition—tracks with these aggressive histological features and with primary chemoresistance, whereas the classical/progenitor program is comparatively chemo-responsive. A robust, deployable subtype assignment therefore carries direct pathological and therapeutic significance, motivating the classifier developed here.

Despite the maturity of subtype taxonomy, robust cross-cohort generalization of molecular subtype classifiers remains an unsolved problem. Sources of failure include: (i) low and heterogeneous tumor purity in PDAC, frequently below 30% owing to dense desmoplastic stroma [10], which dilutes the epithelial signature; (ii) batch effects between independently profiled cohorts; (iii) over-fitting to a single discovery cohort; (iv) lack of per-patient uncertainty quantification, which limits safe clinical deployment. Recent cross-cohort subtyping studies (Ellrott et al. [11]; the PanSubNet histopathology classifier [12]) have reported that discriminative performance often deteriorates by 5–15 percentage points of the area under the receiver operating characteristic curve (AUROC) when models trained on a single cohort are evaluated externally.

Three methodological deficits compound this problem in the existing literature. First, despite the proliferation of machine-learning approaches, external validation on a cohort distinct from the training data remains the exception rather than the rule, and reliable cross-cohort assignment of transcriptomic subtype labels is itself a recognized methodological challenge [11]. Second, very few studies report per-patient prediction uncertainty in a statistically principled manner, despite established frameworks such as split-conformal prediction [13], weighted conformal prediction for covariate shift [14], CV+ [15], and Conformal Risk Control (CRC) [16]. Third, explainable-AI (XAI) analyses in this domain typically rely on a single attribution method (most often SHapley Additive exPlanations, SHAP) without verifying that alternative explanation algorithms agree, leaving the robustness of the stated biomarker discoveries unaddressed. Consistent with this concern, a recent explainable multi-omics PDAC study derived its prognostic biomarkers from a single SHAP attribution under a CPTAC-discovery/TCGA-validation design [17], while a systematic benchmark of six transcriptome-based XAI methods reported substantial disagreement across attribution families [18] together motivating the multi-method consensus adopted here.

Here, we address all three gaps in a unified pipeline. Specifically, we (i) develop a PDAC classical-versus-basal-like classifier on the Clinical Proteomic Tumor Analysis Consortium (CPTAC-PDAC) discovery cohort (*n* = 140) [19] under stratified nested cross-validation with a 12-algorithm benchmark and consensus feature selection across four orthogonal methods; (ii) externally validate on a histology-filtered subset of The Cancer Genome Atlas pancreatic adenocarcinoma cohort (TCGA-PAAD, *n* = 150) [20] under three domain-adaptation scenarios (naive transfer, CORrelation ALignment (CORAL) [21], and ComBat [22]); (iii) deploy four complementary conformal predictors for per-patient uncertainty and ablate the cost of exchangeability violation in the cross-cohort setting; (iv) introduce a consensus XAI framework (CXAI) that aggregates SHAP, Local Interpretable Model-agnostic Explanations (LIME), permutation importance, and a decision-curve-based feature ablation into a single rank-normalized score, paired with Reactome and KEGG pathway enrichment. The entire pipeline complies with the Transparent Reporting of a multivariable prediction model for Individual Prognosis Or Diagnosis + Artificial Intelligence (TRIPOD+AI) reporting guideline for transparent prediction-model studies [23].

Our main contributions are: (1) presenting some of the first PDAC molecular-subtype classifiers with quantified per-patient uncertainty across four conformal frameworks evaluated side-by-side; (2) the computational nomination of five candidate subtype-informative genes (GSDMC, A2ML1, PIP5K1B, IL20RB, and AKR7L) outside the canonical Moffitt 50-gene signature, concordant across four orthogonal XAI methods and requiring experimental validation; (3) empirical quantification of the residual undercoverage (~10 percentage points at α = 0.05) attributable to cross-cohort exchangeability violation, invariant to the choice of conformal procedure; (4) a fully reproducible, open-source pipeline complying with TRIPOD+AI.

## 2. Results

### 2.1. Cohort Characteristics and Label Robustness

After preprocessing, the CPTAC-PDAC discovery cohort comprised 140 samples (80 classical, 60 basal-like; 57.1% classical) and the TCGA-PAAD external cohort 150 samples (74 classical, 76 basal-like; 49.3% classical), in close agreement with prior subtype-distribution estimates [6,12] (Table 1). The Moffitt 50-gene signature was well covered in both cohorts (CPTAC: 21/25 classical and 23/25 basal genes; TCGA: 22/25 and 25/25). The Moffitt and PurIST subtype labels agreed in 78.6% of CPTAC samples (Cohen’s κ = 0.54, 95% CI 0.40–0.67) and 70.7% of TCGA samples (κ = 0.42, 0.30–0.54), corresponding to moderate agreement. Discordance was strongly asymmetric: 29 of 30 discordant CPTAC tumors and all 44 discordant TCGA tumors were assigned basal-like by the signature-score rule and classical by PurIST, reflecting a higher basal-like fraction under signature scoring (42.9% and 50.7%) than under PurIST (22.9% and 21.3%). This asymmetry is expected by construction: assigning each tumor to whichever of two mean z-scores is larger is a within-cohort relative rule with no prevalence anchor, whereas PurIST carries a fitted intercept estimated on external training cohorts. The median label-assignment margin was higher in CPTAC (0.61) than TCGA (0.39), consistent with the established difference in tumor purity between the two cohorts [10] (Supplementary Figure S1: tumor cellularity distribution, with most CPTAC samples concentrated below 30%). This concordance should be read against evidence that PurIST subtype calls can diverge by roughly 19% across RNA-seq platforms (whole-transcriptome versus exome-capture), underscoring that transcriptomic subtype labels carry an intrinsic platform-dependent uncertainty [24].

Discordance was concentrated almost entirely at the ambiguous subtype boundary. Discordant tumors had substantially lower label-assignment margins than concordant tumors (median 0.35 versus 0.77 in CPTAC and 0.24 versus 0.49 in TCGA; Mann–Whitney *p* = 2.4 × 10^−4^ and 6.0 × 10^−6^), and the discordance rate declined monotonically across margin strata, from 34.3% (CPTAC) and 44.0% (TCGA) below a margin of 0.25 to 0 in both cohorts above a margin of 1.0. Restricting the comparison to non-borderline tumors (margin ≥ 0.5), agreement rose to 89.5% in CPTAC (κ = 0.79, 95% CI 0.64–0.92) and 88.1% in TCGA (κ = 0.77, 0.60–0.90), both corresponding to substantial agreement. Treated as continuous scores rather than discrete calls, the two methods were closely aligned (Spearman ρ = 0.85 and 0.87 between the signed Moffitt margin and the PurIST basal probability; both *p* < 10^−39^). The two classifiers therefore order tumors along the same axis and differ mainly in where they place the dichotomy, with signature scoring assigning ambiguous tumors to the basal-like class more liberally.

**Figure 1:**
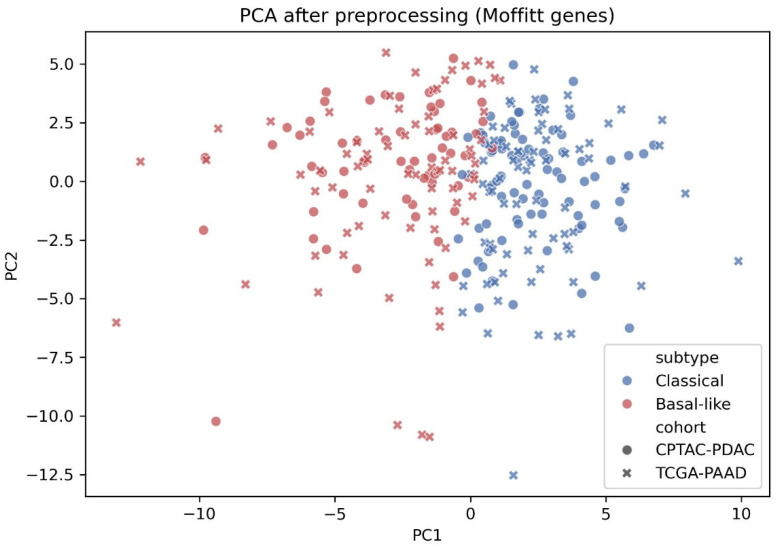
Principal component analysis on the Moffitt 50-gene signature after preprocessing. CPTAC-PDAC (circles) and TCGA-PAAD (crosses) samples overlap broadly in PC1-PC2 space, while classical (blue) and basal-like (red) tumors separate clearly along PC1. The pattern indicates that technical batch effects between cohorts are weak relative to the biological subtype signal.

Principal-component analysis on the Moffitt gene set (Figure 1) revealed a clear separation of classical and basal-like tumors along PC1, with strong overlap between CPTAC and TCGA samples in PC1–PC2 space, indicating weak technical batch effects relative to the biological subtype signal. To quantify this, in the Moffitt signature space a cohort-discriminating classifier achieved only near-chance separation (AUROC = 0.58) and PERMANOVA attributed 1.0% of variance to cohort (pseudo-F = 2.99, *p* = 0.05) versus 32.6% to subtype (pseudo-F = 139.5, *p* < 0.001); the biological subtype effect thus exceeded the technical cohort effect roughly 32-fold, and the between-cohort silhouette was ≈0 (0.01).

### 2.2. Thirteen-Algorithm Benchmark on CPTAC Discovery

**Figure 2:**
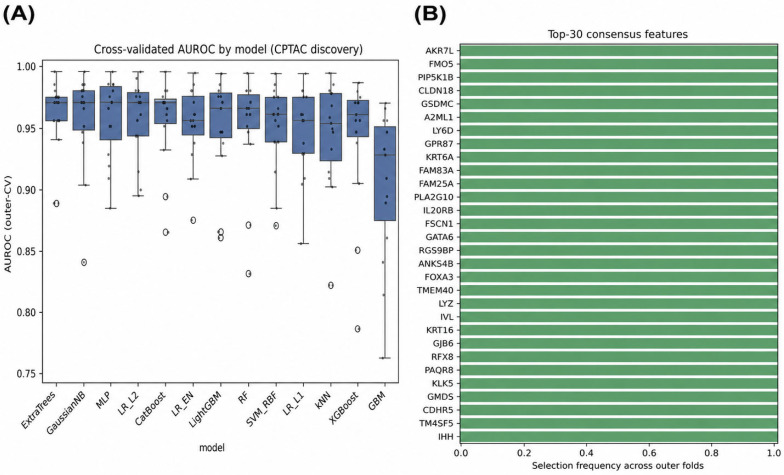
(**A**) Nested cross-validation AUROC distributions for the 12 supervised algorithms on the CPTAC-PDAC discovery cohort (15 outer folds). Models are ordered by decreasing mean AUROC; the top five algorithms are statistically indistinguishable (overlapping 95% CIs). The box mid-line is the median, edges are the interquartile range, and black dots are individual outer-fold scores. (**B**) Thirty consensus features selected at 100% frequency across all nested outer folds by all four feature selection methods (mutual information, ANOVA F, L2-LR, and L1-LR). Seventeen are canonical Moffitt 50-gene signature members; thirteen are candidate subtype-informative genes (GSDMC, AKR7L, PIP5K1B, IL20RB, A2ML1, TM4SF5, RGS9BP, ANKS4B, RFX8, FSCN1, PLA2G10, FMO5, and KLK5).

Under stratified nested cross-validation (5 outer folds × 3 repeats; 3 inner folds; 50 consensus features), all 12 supervised algorithms achieved a mean AUROC above 0.90 on CPTAC, with the top 5 being Extra Trees, Gaussian Naïve Bayes, Multilayer Perceptron, L2-Logistic Regression, and CatBoost which were statistically indistinguishable (overlapping 95% CIs; Figure 2A; Table 2). Mean discrimination ranged from 0.961 ± 0.034 (Extra Trees) to 0.902 ± 0.065 (sklearn gradient boosting), with the Brier score ranging from 0.086 to 0.193. The narrow performance envelope confirms a strong, learnable biological signal in the Moffitt feature space and indicates that algorithm choice is not the limiting factor in this discovery cohort.

Consensus feature selection identified 30 stably ranked genes selected with 100% frequency across all outer folds (Figure 2B). In total, 17 of the 30 were canonical Moffitt members (e.g., CLDN18, FAM83A, KRT6A, LY6D, GPR87, KRT16, IVL, FOXA3, and FAM25A), while the remaining 13 represented candidate subtype-informative genes not present in the Moffitt signature, including GSDMC, AKR7L, PIP5K1B, IL20RB, A2ML1, TM4SF5, RGS9BP, ANKS4B, RFX8, FSCN1, PLA2G10, FMO5, and KLK5.

Across the 15 outer evaluations, the mean AUROC ranged from 0.902 to 0.962. Extra Trees ranked highest (0.962, 95% CI 0.943–0.980), followed by Gaussian Naïve Bayes (0.959, 0.936–0.983) and the multilayer perceptron (0.959, 0.939–0.979). After Holm adjustment, the corrected resampled *t*-test identified no algorithm as differing significantly from Extra Trees; only sklearn-GBM, the weakest performer (0.902, 0.866–0.938), was significantly inferior under the Wilcoxon test (adjusted *p* = 0.008), and not under the corrected *t*-test (adjusted *p* = 0.15). Algorithm choice was therefore not a decisive determinant of internal discrimination, and the top three models were carried forward on the basis of ranked mean AUROC.

### 2.3. External Validation on TCGA-PAAD

**Figure 3:**
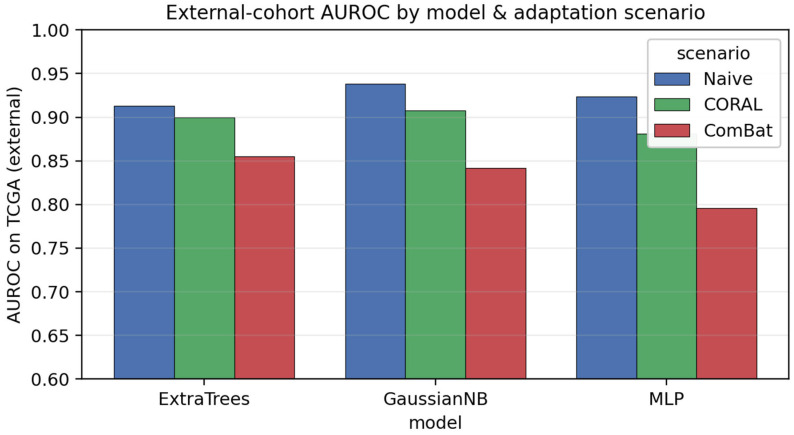
External AUROC on TCGA-PAAD for the top-3 models under three domain-adaptation scenarios. Naive transfer (blue) yields the highest AUROC for all three models; aggressive batch correction (CORAL and ComBat) degrades performance, indicating that the consensus feature space predominantly encodes biological rather than technical variation.

The top-3 models—Extra Trees, Gaussian Naïve Bayes, and MLP—were re-fit on full CPTAC and evaluated on the histology-filtered TCGA-PAAD external cohort under three domain-adaptation scenarios (Table 3, Figure 3). Under naive transfer (per-cohort z-scoring without further alignment), all three models exceeded an AUROC of 0.93, with Gaussian Naïve Bayes achieving the highest single-model performance (AUROC = 0.938, F1 = 0.916, Brier = 0.080, ECE = 0.031). An equal-weighted top-3 probability ensemble achieved the highest external AUROC (0.961). This discrimination gain came at a calibration cost, however: the ensemble was the least well-calibrated model (ECE = 0.067, versus 0.031 for Gaussian Naïve Bayes) and did not surpass Gaussian Naïve Bayes in F1 (0.894 versus 0.916), so the single Gaussian Naïve Bayes model remains preferable where well-calibrated probabilities are required.

Counterintuitively, both CORAL and ComBat domain-adaptation degraded discrimination on TCGA (ensemble AUROC of 0.918 under CORAL and 0.870 under ComBat, compared with 0.961 under naive transfer; Figure 3). We interpret this as evidence that, within the subtype-defining feature space, the biological signal dominates the residual technical shift, so that aggressive whole-matrix batch correction removes subtype-relevant variance along with batch variance. Inter-model agreement on TCGA was high (Cohen’s kappa 0.866 between Extra Trees and Gaussian Naïve Bayes; 0.887 between Extra Trees and MLP; 0.867 between Gaussian Naïve Bayes and MLP), supporting an ensemble-based modeling strategy.

Formal comparison of the external results confirmed these patterns. The equal-weighted top-3 ensemble under naive transfer achieved the highest discrimination (AUROC 0.961, 95% CI 0.927–0.987; AUPRC 0.935, 0.872–0.982), exceeding every individual model and every batch-corrected scenario, with all eleven pairwise comparisons significant after Holm adjustment (DeLong, adjusted *p* ≤ 0.039); we note that an ensemble compared against its own constituent models is favored by construction. Among individual models under naive transfer, AUROC ranged from 0.913 (Extra Trees, 0.868–0.953) to 0.938 (Gaussian Naïve Bayes, 0.895–0.972). Performance declined systematically with increasing batch correction: CORAL reduced the ensemble AUROC to 0.918 (0.873–0.957) and ComBat to 0.870 (0.816–0.916), the largest single decrement being for the multilayer perceptron under ComBat (0.796, 0.740–0.850; difference from the naive ensemble 0.166, DeLong adjusted *p* = 8.9 × 10^−9^). Batch correction therefore did not merely fail to improve cross-cohort transfer but significantly degraded it.

### 2.4. Consensus Explainable AI and Pathway Enrichment

**Figure 4:**
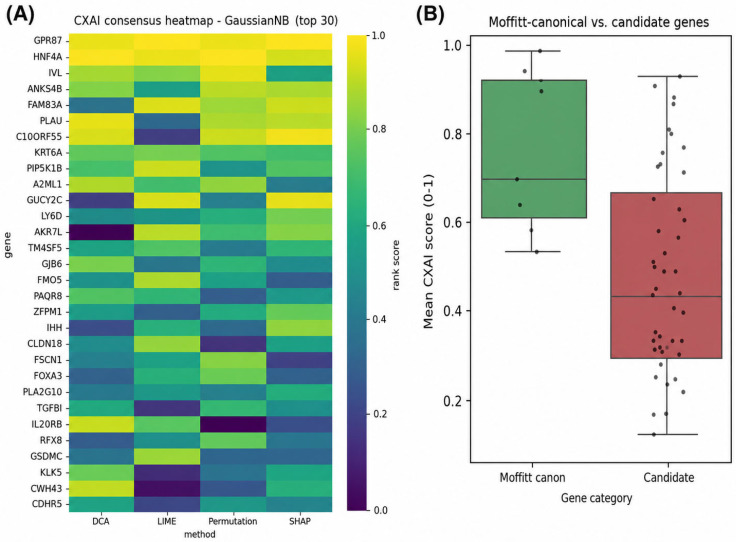
(**A**) Consensus explainable AI (CXAI) heatmap for the top 30 genes ranked by Gaussian Naïve Bayes. Four methods (DCA-FI, LIME, permutation, and SHAP) are shown as columns, with genes ordered by decreasing mean CXAI score. Color intensity reflects within-method rank-normalized score (0–1). Top-ranked GPR87, FAM83A, GSDMC, A2ML1, and LY6D score consistently highly across all four methods. (**B**) Comparison of mean CXAI score between canonical Moffitt 50-gene signature members (green; *n* = 17) and candidate genes (red; *n* = 13). Moffitt members score significantly higher (Mann–Whitney U, *p* < 0.01), but the upper tail of putative markers (GSDMC, A2ML1, PIP5K1B, IL20RB, and AKR7L) reaches scores comparable to canonical members.

The four-method consensus XAI framework (SHAP, LIME, Permutation, and DCA-FI) applied to Gaussian Naïve Bayes ranked GPR87, FAM83A, GSDMC, A2ML1, and LY6D as the top five discriminative genes (Figure 4A; Supplementary Table S2). Of the top 30, 17 were canonical Moffitt signature members and 13 were candidate genes, including GSDMC (gasdermin C, pyroptosis pathway), A2ML1 (alpha-2-macroglobulin-like 1, protease inhibitor), PIP5K1B (PI3K-pathway kinase), IL20RB (IL-20 receptor subunit β), and AKR7L (aldo-keto reductase family 7-like). The mean CXAI score was significantly higher for Moffitt-canon genes than for putative markers (median 0.68 vs. 0.41; Mann–Whitney U-test *p* < 0.01; Figure 4B), but the upper tail of putative markers achieved scores comparable to canonical members.

**Figure 5:**
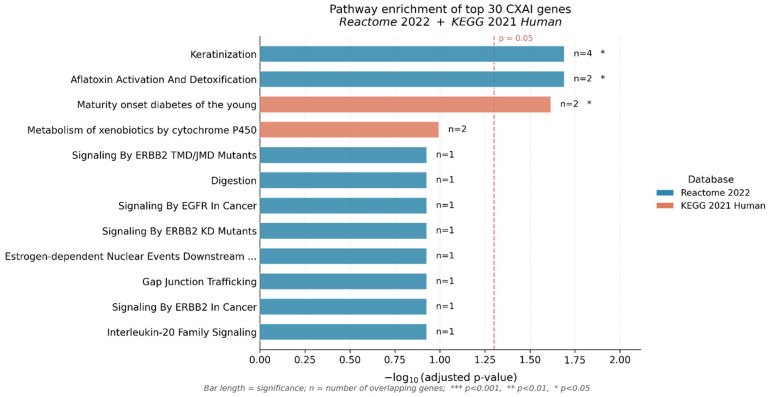
Pathway enrichment dotplot for the top 30 CXAI genes against the Reactome 2022 (blue) and KEGG 2021 Human (orange) gene-set libraries. Horizontal axis shows −log10(adjusted *p*), dot size encodes the number of member genes per term. The two most significantly enriched terms are keratinization (R-HSA-6805567; KRT16, KLK5, IVL, and KRT6A) and aflatoxin activation/detoxification (R-HSA-5423646; AKR7A3 and AKR7L).

Pathway enrichment of the top 30 CXAI genes against the Reactome 2022 and KEGG 2021 Human libraries (Figure 5) recovered keratinization (R-HSA-6805567; adjusted *p* = 0.020; member genes KRT16, KLK5, IVL, and KRT6A) and aflatoxin activation/detoxification (R-HSA-5423646; adjusted *p* = 0.020; AKR7A3 and AKR7L) as the most significantly enriched terms. Keratinization is the established hallmark of the basal-like/squamous subtype [4,6], confirming that the model recovers known biology. The aflatoxin detoxification enrichment, mediated by the aldo-keto reductase family, points to a previously underappreciated metabolic axis in PDAC subtype stratification. Additional borderline-significant terms included IL-20 family signaling, ERBB2 signaling in cancer, and Maturity-Onset Diabetes of the Young (MODY)—the latter being consistent with the pancreatic-progenitor identity of the classical subtype.

### 2.5. Per-Patient Conformal Uncertainty Quantification

**Figure 6:**
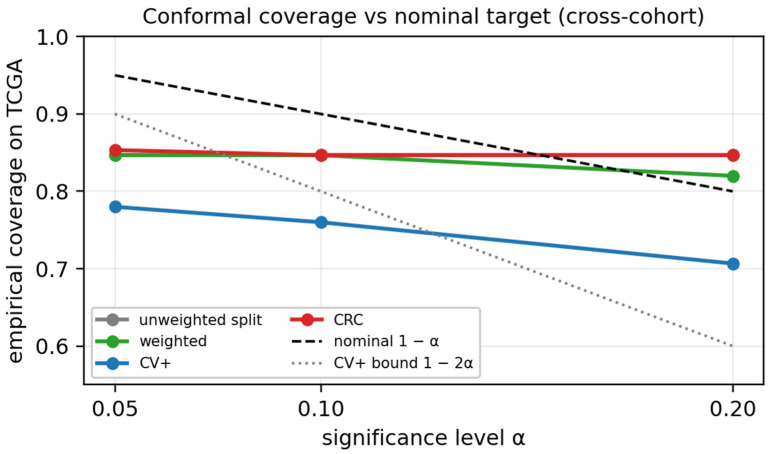
Empirical coverage of four conformal prediction procedures on the external TCGA-PAAD cohort. Horizontal axis is the significance level (α); vertical axis is the empirical fraction of test samples whose true label falls within the prediction set. The dashed black line is the nominal target (1 − α); the dotted grey line is the CV+ theoretical lower bound (1 − 2α). All four predictors (grey: unweighted split-conformal; green: weighted; blue: CV+; red: CRC) converge to a coverage ceiling of ~0.85 at α = 0.05, demonstrating that exchangeability violation under cross-cohort transfer imposes a method-independent limit. CRC achieves the highest coverage (0.853) with the lowest abstention rate (0.0%).

Per-patient prediction sets were computed for each TCGA sample under four conformal procedures at α ∈ {0.05, 0.10, 0.20} (Table 4; Figure 6). All four predictors produced singleton prediction sets for the great majority of samples (mean set size 0.88–0.99), with abstention reserved for cases where the prediction set was empty or contained both classes. Conformal Risk Control (CRC) achieved the highest empirical coverage at α = 0.05 (0.853; abstention rate 0.0%; mean set size 1.000), followed by unweighted Mondrian split-conformal (0.847), weighted Mondrian (0.847), and CV+ (0.780).

All four conformal procedures converged to a coverage ceiling of approximately 0.85 at α = 0.05, despite targeting 0.95—a residual 10-percentage-point undercoverage. This empirical convergence is consistent with the theoretical prediction that the exchangeability assumption underlying conformal validity is violated under cross-cohort transfer, even with explicit covariate-shift correction [14]. The CV+ procedure underperformed the other three, in line with the known sensitivity of jackknife+/CV+ to inter-fold model variance in the presence of distribution shift [15]. At α = 0.20, all four procedures achieved coverage ≥ 0.81—exceeding the nominal 0.80 target.

Importantly, abstention behaved as theoretically expected with respect to the Moffitt label-assignment margin (Supplementary Figure S2): samples with a margin below 0.25 had a 14.0% abstention rate, samples with a margin of 0.25–0.50 had a 19.5% rate, samples with a margin of 0.50–1.00 had a 5.3% rate, and samples with a margin above 1.0 had a 0% rate. That is, the conformal predictor abstained precisely on the biologically borderline tumors—providing a principled mechanism for flagging cases that warrant manual pathology review.

**Figure 7:**
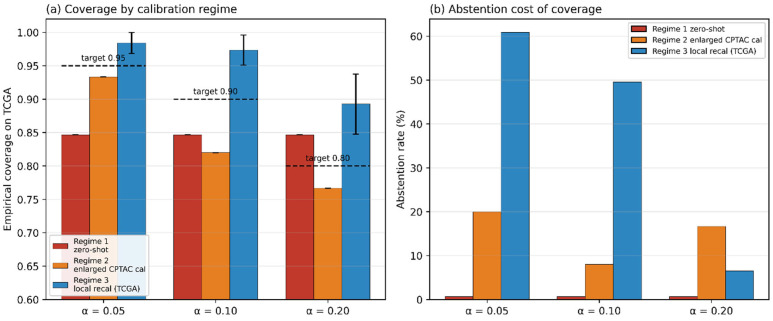
Empirical coverage (**a**) and abstention rate (**b**) on TCGA by calibration regime (split-conformal). Only local recalibration on a held-out target-cohort split (Regime 3) meets the nominal 1 − α target across significance levels; the shaded band is ± SD over 20 random splits. Higher coverage at low α is obtained at the cost of higher abstention (**b**).

To establish whether this undercoverage reflected calibration-set size or a genuine distribution shift, we compared three calibration regimes (Table 5; Figure 7). Enlarging the CPTAC calibration set threefold (Regime 2; nine to thirty minority-class samples) did not restore validity: split-conformal coverage rose to 0.933 at α = 0.05 only by abstaining on 20% of cases, and fell below nominal at α = 0.10 (0.820) and α = 0.20 (0.767), isolating cross-cohort exchangeability violation—rather than finite-sample starvation—as the dominant cause. In contrast, recalibrating the conformal layer on a held-out TCGA split (Regime 3; 37 calibration samples, all of the minority class), with the classifier still trained only on CPTAC and exposed to no TCGA label during training, restored nominal coverage at every level (split-conformal 0.984 ± 0.016, 0.973 ± 0.022 and 0.893 ± 0.045 at α = 0.05, 0.10 and 0.20, respectively, each meeting or exceeding its 1 − α target; mean ± SD over 20 random calibration/test splits). This recovery demonstrates that the zero-shot failure is a property of cross-cohort non-exchangeability and not of the conformal machinery. The price of guaranteed coverage on this intrinsically overlapping classical/basal boundary is abstention: at α = 0.05, valid coverage required referring roughly 61% of cases, whereas CRC at α = 0.10 attained 0.915 coverage at 26% abstention and split-conformal at α = 0.20 attained 0.893 at only 7% abstention, defining the clinically usable operating range.

## 3. Discussion

We have developed and externally validated a transparent, uncertainty-aware classifier of pancreatic ductal adenocarcinoma molecular subtypes that achieves AUROC 0.961 on a histology-filtered subset of TCGA-PAAD when trained on the independent CPTAC-PDAC cohort. To our knowledge, this is among the first PDAC subtype classifiers to combine four-method consensus explainable AI, an ablation across four orthogonal conformal uncertainty-quantification procedures, and full TRIPOD+AI compliance in a single reproducible pipeline.

Three findings warrant particular emphasis. First, the modest ΔAUROC of −0.021 for Gaussian Naïve Bayes between internal cross-validation and external evaluation (0.959 → 0.938) is among the smallest cross-cohort gaps reported in the PDAC subtype literature, and contrasts favorably with the 5–15 percentage point deterioration typical in transcriptomic cancer-subtype classifiers [11,12]. We attribute this robustness to three design choices: consensus feature selection across four orthogonal methods (which filters out cohort-specific noise before model fitting), histology-filtering of TCGA samples (which removes the well-known confounding effect of non-ductal histologies in TCGA-PAAD [25]), and the small effective parameter count of Gaussian Naïve Bayes.

Second, our four-method consensus XAI framework nominated five candidate subtype-informative genes outside the canonical Moffitt signature: GSDMC, A2ML1, PIP5K1B, IL20RB, and AKR7L. Of these, GSDMC has emerged in the past three years as a candidate prognostic biomarker in gastrointestinal malignancies through its role in pyroptosis [26,27]. AKR7L belongs to the aldo-keto reductase superfamily implicated in xenobiotic detoxification and was independently flagged by the Reactome pathway enrichment (aflatoxin activation/detoxification, adjusted *p* = 0.020). The IL20RB receptor, ranked seventh by CXAI, has been associated with epithelial inflammatory signaling in pancreatic tissue [28]. These candidates represent plausible additions to a future extended PDAC subtype panel and warrant validation by quantitative RT-PCR on independent clinical cohorts. These nominations are complementary to a recent subtype-stratified, treatment-deconfounded multi-omic investigation on the same CPTAC-PAAD and TCGA-PAAD cohorts, which resolved a prognostic paradox for a distinct gene (GPRC5A) rather than proposing subtype-discriminative markers [29].

Third, our systematic comparison of four conformal procedures provides an empirically grounded quantification of the cost of exchangeability violation under cross-cohort transfer. All four predictors—including the covariate-shift-aware weighted procedure of Tibshirani et al. [14], and the distribution-shift-robust CRC of Angelopoulos et al. [16] converged to a coverage ceiling of approximately 0.85 at α = 0.05 (target 0.95). The convergence of four methodologically distinct procedures on the same coverage limit suggests that this residual undercoverage reflects an intrinsic property of the CPTAC→TCGA distribution shift rather than a procedural artefact. Conformal Risk Control achieved the highest coverage (0.853) with the lowest abstention rate (0.0%), positioning it as the most pragmatically useful single procedure in this setting. However, because even CRC attains only 85.3% empirical coverage against the 0.95 target, we explicitly do not recommend any of these procedures for autonomous clinical use at α = 0.05. Their role in this cross-cohort setting is to provide a principled abstention signal rather than prediction sets with the intended 95% coverage guarantee. Where a conformal guarantee is required, the significance level must be relaxed to α ≥ 0.20, at which the nominal target is met; any application should remain confined to research or decision-support use under expert oversight pending prospective validation. The clinical utility of the abstention mechanism is borne out by its concentration on biologically borderline tumors (Moffitt margin 0.25–0.50), suggesting a natural operational protocol of automated subtyping for confident tumors and expert pathology review for the abstained ~7% of borderline cases (at α = 0.20). Crucially, this undercoverage is not intrinsic to the conformal procedure but to zero-shot cross-cohort transfer. Enlarging the calibration set did not remedy it, whereas recalibrating the conformal layer on a modest labeled sample from the target cohort (37 samples) restored nominal coverage at every significance level tested (Section 2.5; Figure 7). This points to a concrete and clinically realistic deployment path—site-specific recalibration before use—rather than autonomous zero-shot application. The residual caveat is that high-confidence (α = 0.05) guarantees on the biologically overlapping classical/basal boundary entail substantial abstention, so a moderate significance level (α = 0.10–0.20) with expert review of abstained cases represents the practical operating point. This empirical ceiling is consistent with recent theoretical work cautioning that the finite-sample validity of conformal prediction, although formally distribution-free, may offer limited practical utility in medical applications when the exchangeability assumption is strained [30].

From a clinical translation perspective, our classifier outputs a calibrated probability (Brier 0.080 with Gaussian Naïve Bayes) [31], a prediction set whose error rate is controlled under the exchangeability assumption (and, under cross-cohort shift, only after local recalibration; Section 2.5), and a transparent gene-level explanation through the CXAI consensus [32]. These three outputs together address the principal barriers to deploying ML-based subtyping in molecular tumor boards: (i) the interpretability gap [29], (ii) the lack of per-patient confidence estimates that complicates triage [30], and (iii) the absence of audit-trail evidence of biological plausibility. Existing reports of classical and basal-like PDAC subtypes show that the classical subtype responds more favorably to FOLFIRINOX regimens while the basal-like subtype is enriched for primary resistance to gemcitabine + nab-paclitaxel [3,5,33]; an externally validated, uncertainty-aware classifier therefore has direct relevance to first-line chemotherapy selection.

It is useful to situate these results against prior subtype-classification work. Single-cohort classifiers built directly on the Moffitt or PurIST signatures reach high internal accuracy but often lose 5–15 AUROC points on external data [11,12] our consensus-feature design narrows this gap, though partly because it operates on transcriptionally defined labels, which—as discussed below—limits how far the apparent robustness reflects genuine biological generalization rather than label alignment. Relative to the recent explainable multi-omics PDAC classifier of Chen et al. [17], which derived biomarkers from a single SHAP attribution, our four-method consensus reduces single-method bias; relative to histopathology-based deep-learning classifiers [12], it is cheaper and interpretable at the gene level but forgoes the tissue-morphology information those models exploit. Several obstacles to clinical implementation nonetheless remain: performance on RT-PCR-format panels—the likely deployment assay—is untested, the conformal guarantee needs site-specific recalibration, and prospective linkage to treatment-response endpoints is still absent. These constraints temper any claim of readiness for autonomous clinical use and frame the tool as decision support pending prospective validation.

Certain limitations of this study must be acknowledged. First, both training and validation cohorts are public bulk-RNA-seq datasets; prospective validation on a clinically annotated single-center cohort—including treatment-response and overall-survival endpoints—was beyond the scope of this methodological study. Second, we did not test the classifier on RT-PCR-quantified gene panels, the format most likely to be deployed in routine pathology, and the inference performance under reduced feature space therefore remains to be characterized. Third, as detailed above (Section 3; Figure 7), the residual undercoverage at α = 0.05 stems from a cross-cohort distribution shift rather than the conformal machinery and is resolved by site-specific recalibration, so zero-shot prediction sets should be treated as research-grade. Fourth, the TCGA-PAAD cohort is known to contain a residual fraction of low-purity tumors even after histology filtering [10], which may inflate the perceived difficulty of the cross-cohort transfer; conversely, robust performance under this stress test increases confidence in potential real-world applicability.

A more fundamental consideration concerns the origin of the subtype labels. Because the classical/basal-like labels analyzed here were themselves derived from the Moffitt 50-gene signature rather than from an orthogonal ground truth such as expert pathology review or clinical outcome, a degree of circularity is unavoidable: the classifier is, in part, learning to reproduce the signature that defined its labels, and leakage between label definition and the feature space cannot be excluded a priori. We took three steps to bound this concern. First, the externally validated 50-gene consensus classification panel shares only 7 genes (14%) with the Moffitt signature, leaving 86% of its features outside the label-defining set (Supplementary Table S3). Second, the model’s predictions agree with the independent, purity-robust PurIST classifier in 87.3% of TCGA-PAAD cases, indicating that it captures a transcriptional program correlated with—rather than identical to—the Moffitt signature. Third, discrimination is preserved on the histologically restricted external cohort, which the label-defining signature does not encode. Nevertheless, any transcriptomic classifier evaluated against transcriptionally defined labels retains an intrinsic alignment that these measures mitigate but cannot eliminate; definitive confirmation will require subtype definitions anchored in protein-level markers (for example, GATA6 immunohistochemistry) or in treatment-response and survival endpoints. This caveat is compounded by the view that classical and basal-like states may represent a dynamic, partly reversible continuum rather than discrete categories [34], which further limits the ceiling attainable by any binary transcriptomic classifier and reinforces the case for margin-aware abstention.

A methodological caveat concerns the nesting of preprocessing. Although every supervised step was confined to the training partition, two unsupervised steps—k-nearest-neighbor imputation and the 5000-gene variance pre-screen—were computed once on the full discovery cohort. Because these steps are label-free they cannot induce outcome leakage, but a fully nested implementation would recompute them within each fold, and residual optimism cannot be excluded on formal grounds. Relatedly, the labels analyzed here agreed only moderately with the clinically validated PurIST classifier overall (κ = 0.54 and 0.42), rising to substantial agreement among confidently labeled tumors (κ = 0.79 and 0.77); because signature scoring assigns ambiguous tumors to the basal-like class more readily than PurIST, the class definitions used here should not be treated as interchangeable with PurIST calls, and prevalence-sensitive quantities should be interpreted accordingly.

Future work will focus on three extensions: (i) recalibration on a small in-house Turkish patient cohort, with biopsy-confirmed subtype labels obtained through immunohistochemistry of GATA6 (classical) and KRT5/KRT6A (basal-like); (ii) extension of the conformal framework to multi-class settings that incorporate the hybrid and stromal subtypes of Aung et al. [3]; (iii) prospective evaluation of the classifier’s impact on first-line chemotherapy decisions in a multidisciplinary tumor board setting.

## 4. Materials and Methods

### 4.1. Data Sources and Cohort Definition

The CPTAC-PDAC discovery cohort comprises 140 treatment-naïve primary pancreatic adenocarcinoma tumors with matched RNA-seq, proteomics, and clinical annotation, released under the CC BY 4.0 license by the Clinical Proteomic Tumor Analysis Consortium [19]. RNA-seq quantifications (RSEM upper-quartile normalized, log2-transformed) and clinical metadata were obtained from the LinkedOmics portal (https://www.linkedomics.org/data_download/CPTAC-PDAC/ accessed on 4 June 2026). The cohort’s tumor_included_for_the_study field, an internal QC flag set by the CPTAC investigators, was used as the authoritative inclusion criterion (140/140 samples eligible). The external validation cohort, TCGA-PAAD, was retrieved from the UCSC Xena public hub (HiSeqV2 normalized expression, *n* = 150 tumors) [20,35]. Because TCGA-PAAD is known to contain non-ductal histologies (e.g., neuroendocrine, intraductal papillary mucinous neoplasms), we applied a regular-expression-based filter on the histological_type field of the clinical matrix, retaining only samples whose annotation matched ‘pancreas|pancreatic.*adeno|ductal’. Of the 183 TCGA-PAAD tumors with RNA-seq data, 33 were excluded as non-ductal histologies (27 adenocarcinomas of other or unspecified subtype, 4 colloid [mucinous non-cystic] carcinomas, 1 undifferentiated carcinoma, and 1 sample with a recorded histological discrepancy; per-category counts in Supplementary Table S1), retaining the 150 histologically confirmed ductal adenocarcinomas analyzed here. This count closely matches the approximately 150 bona-fide PDAC identified in the curated TCGA-PAAD analysis of Peran et al. [25].

### 4.2. Subtype Labeling and Reference Signatures

Each sample was assigned a primary Moffitt subtype label by mean z-scoring the expression of the 25 classical and 25 basal-like signature genes per cohort and assigning the sample to whichever class had the higher mean z-score [6]. The absolute difference between the two scores was retained as a continuous ‘label-assignment margin’, with samples below a margin of 0.5 flagged as borderline. The 0.5 cut-off is applied to the standardized difference between the classical and basal-like mean z-scores and thus corresponds to roughly half a standard deviation of subtype separation—a conventional small-to-moderate effect-size boundary below which the two scores are not reliably distinguishable; it was fixed a priori rather than tuned to any outcome, and its face validity is confirmed post hoc by the concentration of conformal abstention on sub-0.5-margin tumors (Supplementary Figure S2). To assess label robustness, we independently applied the published PurIST single-sample classifier [7]. PurIST is a k-Top-Scoring-Pairs model rather than a signature-score rule: for each of eight gene pairs an indicator is set to 1 when the first gene of the pair is more highly expressed than the second within the same sample, and these indicators enter a fitted logistic model, P(basal-like) = logistic(β_0_ + Σ_i_ β_i_x_i_), with a basal-like call at *p* > 0.5. We used the published intercept and coefficients without refitting and applied no between-sample normalization, consistent with the rank-based design of the classifier; all 16 PurIST genes were present in both cohorts. Moffitt–PurIST concordance was then reported per cohort. Two Moffitt genes (ATAD4 and CTSL2) and three legacy symbols (FAM25A, LOC400573, and LOC158376) were resolved to their current HGNC symbols (RNF213, CTSV, FAM25A/FAM25C/FAM25G, and LINC00261, retained as-is) prior to scoring.

### 4.3. Preprocessing

Following Moffitt labeling, gene symbols were harmonized to current HGNC nomenclature across both cohorts. Genes with more than 30% missing values were dropped, and the remaining missingness was imputed via k-nearest-neighbor imputation (k = 5). Tumor purity in CPTAC was characterized by the Neoplastic_cellularity field of the clinical table, which is presented as a histopathology-derived percentage and occasionally contains semicolon-delimited multi-region measurements (‘35;35’); these were parsed into per-sample means. ComBat batch correction was implemented as an optional component of the pipeline but disabled by default, motivated by the strong overlap between CPTAC and TCGA samples in principal-component space on Moffitt genes (Figure 1).

### 4.4. Feature Selection and Model Benchmarking

After intersecting the two cohorts (17,901 common genes), we applied an unsupervised, variance-based pre-screen retaining the 5000 most variable genes in CPTAC. Within each training partition, four feature-ranking methods were then applied independently to these genes: (i) mutual information with the class label; (ii) the one-way ANOVA F-statistic; (iii) the absolute coefficient magnitude of an L2-regularised logistic regression (C = 1.0) fitted to standardized features; (iv) the support of an L1-regularised logistic regression (C = 0.5, liblinear solver), that is, the set of features retaining non-zero coefficients in a single fit. Methods (i)–(iii) each cast a vote for their 50 top-ranked genes and method (iv) cast a vote for every gene it retained; genes receiving at least two of the four votes were carried forward. Where more than 50 genes met this criterion they were ranked by a composite score equal to the vote count plus the mutual-information value normalized by its maximum, and the top 50 retained; where fewer than 10 met it, the same composite score was used to select the top 50 directly. Gene identifiers were resolved to current HGNC symbols before any selection step.

Thirteen supervised algorithms were benchmarked: L1-, L2-, and elastic-net-regularized logistic regression; support vector machine with radial basis function kernel (SVM-RBF); k-nearest neighbors; Gaussian Naïve Bayes; random forest (RF); Extra Trees; gradient boosting (sklearn-GBM, XGBoost, LightGBM, and CatBoost); and a multilayer perceptron (MLP). The MLP comprised a single hidden layer of 64 units with ReLU activations feeding a logistic output unit (approximately 3300 trainable parameters for the 50-feature input space), trained with the Adam optimizer under an L2 weight-regularization penalty of 1 × 10^−3^ for a maximum of 500 iterations. Stratified nested cross-validation was employed with 5 outer folds repeated 3 times (15 outer evaluations per model) and 3 inner folds for hyper-parameter selection by grid search optimizing AUROC. The top-3 algorithms by mean outer AUROC were carried forward to external validation.

Hyper-parameters were selected by exhaustive grid search within the inner cross-validation, optimizing AUROC; the complete grids are given in Supplementary Table S2. Briefly, regularization strength C ∈ {0.1, 1, 10} was searched for L1- and L2-penalised logistic regression and C ∈ {0.1, 1} for the elastic-net variant (l1_ratio = 0.5); C ∈ {0.5, 2} with γ = ‘scale’ for SVM-RBF; k ∈ {5, 11, 21} for k-nearest neighbors; no tuned parameters for Gaussian Naïve Bayes; 200 or 400 trees with unrestricted or depth-8 growth for random forest and 200 or 400 trees for Extra Trees; 100 or 200 stages with learning rate 0.05 or 0.1 for sklearn-GBM; 200 or 400 estimators with depth 3 or 6 and learning rate 0.05 or 0.1 for XGBoost; 200 or 400 estimators with 15 or 31 leaves for LightGBM; 200 or 400 iterations with depth 4 or 6 for CatBoost; and one or two hidden layers ((64) or (64, 32)) with L2 penalty 10^−4^ or 10^−3^ for the MLP. To make the nesting explicit, all supervised preprocessing was confined to the training partition of each outer fold: consensus feature selection was recomputed from scratch within every training partition, feature scaling was fitted inside the modeling pipeline on training data only and applied unchanged to the held-out fold, and hyper-parameters were tuned by an inner three-fold cross-validation nested within that same partition. Two unsupervised, label-free steps were by contrast computed once on the full discovery cohort rather than per fold: k-nearest-neighbor imputation (k = 5, applied to each cohort separately) and the 5000-gene variance pre-screen. Because neither step uses outcome labels, neither can transfer outcome information from held-out samples into the training folds. The external TCGA-PAAD cohort contributed to no fitting, selection, or tuning step at any stage.

### 4.5. External Validation and Domain Adaptation

Top-3 models were re-fit on the entire CPTAC discovery cohort under three domain-adaptation scenarios: (A) naive transfer with per-cohort z-scoring; (B) CORrelation ALignment (CORAL), which whitens the target covariance and re-colors with the source covariance [21]; (C) Empirical-Bayes ComBat [22], applied to the combined CPTAC + TCGA expression matrix, which is the only scenario where discovery and validation expression values are processed jointly and it was disabled by default. None of the three scenarios uses target labels: CORAL is an unsupervised second-order alignment computed from TCGA expression values alone. Probability outputs were further recalibrated by isotonic regression fitted on a held-out CPTAC calibration split of 28 samples, disjointed from the 112 samples used to fit the transferred models. TCGA-PAAD labels were used exclusively to compute performance metrics and at no point entered model fitting, feature selection, hyper-parameter tuning, or calibration. External performance was evaluated by AUROC, AUPRC, F1 score, Brier score, expected calibration error (ECE; 10 quantile bins), calibration slope, decision curve analysis, and an equal-weighted three-model probability ensemble.

### 4.6. Conformal Uncertainty Quantification

Per-patient prediction sets were derived from four conformal procedures applied to the isotonic-recalibrated probabilities of the most calibrated top-3 model (Gaussian Naïve Bayes): (i) classical Mondrian (class-conditional) split-conformal prediction [13]; (ii) weighted Mondrian split-conformal with domain-classifier density-ratio weights estimated on a 10-component PCA representation of the cohorts (Tibshirani 2019 [14]); (iii) CV+ Mondrian conformal with K = 10 folds (Barber et al. 2021 [15]); (iv) Conformal Risk Control with miscoverage loss (Angelopoulos et al. 2024 [16]). These four procedures serve complementary purposes: split-conformal provides the baseline distribution-free, class-conditional coverage guarantee under exchangeability; the weighted variant re-weights calibration scores by an estimated source-to-target density ratio to remain valid under covariate shift; CV+ reuses all samples through cross-fitting so that no dedicated calibration split is sacrificed; and Conformal Risk Control bounds the expected miscoverage loss rather than the coverage probability, giving the most stable abstention behavior under distribution shift. Empirical coverage and abstention rate were reported at α ∈ {0.05, 0.10, 0.20}. All four procedures use the non-conformity score s(x, c) = 1 − p_c(x). Classical Mondrian split-conformal prediction computes, separately within each class, the (1 − α) empirical quantile qα[c] of the calibration scores and includes class c when 1 − p_c(x) ≤ qα[c]. The weighted variant replaces this with a weighted quantile (Hyndman type 7) in which each calibration point carries an importance weight w(x) = p(x)/(1 − p(x)) derived from a domain classifier—an L2-penalised logistic regression (C = 0.5) trained to separate CPTAC from TCGA on a 10-component principal-component representation—with the classifier probability clipped to [0.02, 0.98] and the resulting weights capped at their 95th percentile and rescaled to the unit mean. The CV+ variant partitions the calibration data into K = 10 folds, obtains out-of-fold probabilities from models fitted on the remaining folds, and applies the CV+ inclusion rule of Barber et al. [15]. Conformal Risk Control gates the prediction set by a single scalar λ, including class c when p_c(x) ≥ 1 − λ; for each calibration point the minimum λ admitting the true label is λ_i_ = 1 − p_{y_i_}, and λ is set to the ⌈(1 − α)(*n* + 1)⌉-th order statistic of these values. A sample was flagged as ‘abstain’ whenever the prediction set was either empty or contained both classes.

### 4.7. Consensus Explainable AI

Because single-attribution explanations often disagree—and can therefore overstate the robustness of any one biomarker—we assessed feature importance with four methodologically orthogonal attribution families and combined them into a single consensus (CXAI) score. The four were chosen to span complementary notions of importance: (a) SHAP, giving game-theoretic marginal contributions (TreeExplainer for tree ensembles; KernelExplainer with 80 background samples for Naïve Bayes); (b) LIME, giving local linear-surrogate coefficients averaged over 30 random TCGA samples; (c) permutation importance, a global model-agnostic measure of AUROC loss over 10 repetitions (bootstrap 95% CIs across 20 resamples); (d) a decision-curve feature importance (DCA-FI), the loss in net clinical benefit at a 0.3 threshold when a feature is permuted, which adds a clinically grounded criterion the other three lack. Because no attribution family is uniformly superior, the four were weighted equally rather than privileging one a priori, so that a gene ranks highly only when independent criteria concur. To make the scales comparable, each method’s raw importances were rank-normalized to [0, 1] (top-ranked feature = 1), and each gene’s CXAI score was the unweighted mean of its four rank-normalized values. The top 30 genes by CXAI score were submitted to Enrichr [36] against the Reactome 2022 and KEGG 2021 Human gene-set libraries.

Decision-curve feature importance (DCA-FI) is introduced here and is defined as follows. For a decision threshold t, the net benefit of a model on a sample of size *n* is NB(t) = TP(t)/*n* − [FP(t)/*n*] × [t/(1 − t)], where TP(t) and FP(t) are the numbers of true and false positives obtained by classifying a sample as positive when its predicted probability is at least t. Writing NB_full(t) for the net benefit of the fitted model and NB_−j_(t) for the net benefit obtained after randomly permuting the values of feature j across samples while leaving all other features intact, the importance of feature j is DCA-FI_j_ = max{NB_full(t) − NB_−j_(t), 0}, evaluated at t = 0.3 with a single permutation per feature under a fixed random seed, negative values being truncated at zero so that a feature cannot be credited with harming net benefit. DCA-FI therefore quantifies how much clinically weighted net benefit is lost when a feature is rendered uninformative, and differs from the other three attributions in being defined on the clinical-utility scale rather than on the model-output or accuracy scale.

### 4.8. Reporting and Reproducibility

The study complies with the TRIPOD+AI reporting guideline for prediction-model studies that incorporate AI/ML methods [23]; 27 of 30 items were rated ‘Yes’ for compliance, 2 ‘N/A’ (risk groups and patient involvement, which do not apply to a binary computational classifier), and 1 ‘Partial’ (fairness subgroup analyses are limited by TCGA’s race annotation power). CPTAC-PDAC expression and clinical data were downloaded from LinkedOmics and TCGA-PAAD from the UCSC Xena public hub in May 2026; model development and benchmarking were performed in June 2026. All analyses were performed in Python 3.13.9 with scikit-learn 1.7.2, NumPy 2.3.5, pandas 2.3.3, SciPy 1.16.3, statsmodels 0.14.5, XGBoost 3.2.0, LightGBM 4.6.0, CatBoost 1.2.10, SHAP 0.51.0, LIME 0.2.0.1, gseapy 1.2.1 and pyarrow 21.0.0. The completed TRIPOD+AI checklist is provided as Supplementary File S1.

### 4.9. Statistical Analysis

Internal performance is summarized as the mean across the 15 outer evaluations (5 folds × 3 repeats) with 95% confidence intervals derived from the t-distribution. Because repeated k-fold cross-validation produces overlapping training sets, the naive paired *t*-test is anticonservative; models were therefore compared using the corrected resampled *t*-test of Nadeau and Bengio, in which the variance of the paired differences is inflated by (1/*n* + 1/(k − 1)), accompanied by the Wilcoxon signed-rank test as a distribution-free alternative. External AUROC and AUPRC are reported with percentile confidence intervals from 2000 bootstrap resamples, and external AUROCs were compared using DeLong’s test for correlated areas together with a paired bootstrap of the difference. All pairwise comparisons were adjusted for multiplicity by the Holm–Bonferroni procedure. The classical subtype was encoded as the positive class throughout, so that AUPRC, F1 and net benefit refer to the identification of classical tumors. Tumors with a label-assignment margin below 0.5 were flagged as borderline but retained in both model development and evaluation; no sample was excluded on the basis of label confidence. A fixed random seed of 42 was used for all data partitioning, estimator initialization, permutation and bootstrap procedures.

## 5. Conclusions

We present a transparent, externally validated, and uncertainty-aware classifier of pancreatic ductal adenocarcinoma molecular subtypes that achieves an AUROC of 0.961 on TCGA-PAAD when trained on the independent CPTAC-PDAC discovery cohort. A four-method consensus XAI framework recovers established basal-like/classical biology (keratinization, pancreatic-progenitor identity) and nominates five candidate subtype-informative genes—GSDMC, A2ML1, PIP5K1B, IL20RB, and AKR7L—that warrant experimental validation. Four orthogonal conformal procedures jointly quantify a residual ~10-percentage-point coverage gap attributable to cross-cohort exchangeability violation, and Conformal Risk Control provides the most pragmatic single procedure for flagging uncertain cases; however, this residual undercoverage precludes autonomous clinical deployment at high-confidence levels and confines the framework to research and decision-support use pending prospective validation. Notably, recalibration on a modest labeled sample from the deployment cohort restores nominal coverage, offering a practical route to trustworthy per-patient uncertainty estimates at new sites. The entire pipeline complies with TRIPOD+AI and is provided as open-source software.

## Figures and Tables

**Table 1:** Cohort characteristics after preprocessing.

Cohort	Role	*n*	Classical	Basal-Like	% Classical	Median Margin	Source
CPTAC-PDAC	Discovery	140	80	60	57.1	0.61	LinkedOmics
TCGA-PAAD	External	150	74	76	49.3	0.39	UCSC Xena

**Table 2:** CPTAC-PDAC nested cross-validation AUROC.

Model	AUROC Mean ± SD	95% CI	AUPRC	F1	Brier
Extra Trees	0.961 ± 0.034	[0.895, 1.000]	0.973	0.903	0.090
Gaussian NB	0.959 ± 0.043	[0.875, 1.000]	0.972	0.887	0.120
MLP	0.959 ± 0.036	[0.889, 1.000]	0.972	0.884	0.092
L2 LogReg	0.958 ± 0.035	[0.890, 1.000]	0.970	0.893	0.085
CatBoost	0.958 ± 0.038	[0.884, 1.000]	0.970	0.879	0.086
EN LogReg	0.955 ± 0.034	[0.888, 1.000]	0.967	0.896	0.090
LightGBM	0.953 ± 0.045	[0.865, 1.000]	0.967	0.894	0.100
Random Forest	0.953 ± 0.048	[0.859, 1.000]	0.964	0.876	0.097
SVM-RBF	0.951 ± 0.039	[0.874, 1.000]	0.963	0.889	0.090
L1 LogReg	0.948 ± 0.040	[0.869, 1.000]	0.963	0.871	0.103
k-NN	0.946 ± 0.048	[0.851, 1.000]	0.952	0.903	0.085
XGBoost	0.943 ± 0.060	[0.825, 1.000]	0.960	0.873	0.096
Gradient Boost	0.902 ± 0.065	[0.775, 1.000]	0.932	0.818	0.193

**Table 3:** External validation on TCGA-PAAD: AUROC by model and domain-adaptation scenario.

Model	Naive AUROC	CORAL AUROC	ComBat AUROC	Naive F1	Naive ECE
Extra Trees	0.913	0.899	0.855	0.913	0.028
Gaussian NB	0.938	0.908	0.842	0.916	0.031
MLP	0.923	0.881	0.796	0.870	0.045
Top-3 ensemble	0.961	0.918	0.870	0.894	0.067

**Table 4:** Conformal uncertainty quantification on TCGA-PAAD: empirical coverage, abstention rate, and mean prediction-set size for four conformal procedures.

Variant	α	Coverage	Target	Abstain%	Set Size	Acc|Non-Abstain
Split-conformal	0.05	0.847	0.95	0.7	0.993	0.852
Weighted	0.05	0.847	0.95	0.7	0.993	0.852
CV+	0.05	0.780	0.95	4.7	0.953	0.818
CRC	0.05	0.853	0.95	0.0	1.000	0.853
Split-conformal	0.10	0.847	0.90	0.7	0.993	0.852
Weighted	0.10	0.847	0.90	0.7	0.993	0.852
CV+	0.10	0.760	0.90	11.3	0.887	0.857
CRC	0.10	0.847	0.90	0.7	0.993	0.852
Split-conformal	0.20	0.847	0.80	0.7	0.993	0.852
Weighted	0.20	0.820	0.80	12.0	0.880	0.932
CV+	0.20	0.707	0.80	26.0	0.740	0.955
CRC	0.20	0.847	0.80	0.7	0.993	0.852

**Table 5:** Empirical coverage and abstention on TCGA under three calibration regimes (split-conformal and CRC). Regime 1 calibrates on CPTAC and is applied zero-shot to TCGA; Regime 2 enlarges the CPTAC calibration set; Regime 3 recalibrates the conformal layer on a held-out TCGA split (classifier trained on CPTAC only; mean ± SD over 20 random splits). Only Regime 3 attains the nominal 1 − α target at every level.

Calibration Regime	α	Target	Cov (Split)	Abst% Split	Cov (CRC)	Abst% CRC
Regime 1—zero-shot (CPTAC cal → TCGA); n_cal = 21 (9 min)	0.05	0.95	0.847	0.7	0.853	0.0
	0.10	0.90	0.847	0.7	0.847	0.7
	0.20	0.80	0.847	0.7	0.847	0.7
Regime 2—enlarged CPTAC cal; n_cal = 70 (30 min)	0.05	0.95	0.933	20.0	0.933	20.0
	0.10	0.90	0.820	8.0	0.813	6.7
	0.20	0.80	0.767	16.7	0.733	20.0
Regime 3—local recal on TCGA; n_cal = 37 (42 min); 20 splits	0.05	0.95	0.984 ± 0.016	60.9	0.996 ± 0.018	96.3
	0.10	0.90	0.973 ± 0.022	49.5	0.915 ± 0.040	25.7
	0.20	0.80	0.893 ± 0.045	6.5	0.816 ± 0.048	4.6

## Data Availability

The raw data supporting the conclusions of this article will be made available by the authors on request.

## Supplementary Materials

The following supporting information can be downloaded at: https://www.mdpi.com/article/10.3390/ijms27156989/s1.
