# Supplementary material for: A Generalizable and Interpretable Framework for Molecular Subtype Classification of Pancreatic Ductal Adenocarcinoma Integrating Conformal Uncertainty Quantification and Consensus-Based Explainable Artificial Intelligence Across Multiple Cohorts
Source: Int J Mol Sci. 2026 Aug 4;27(15):6989. doi: 10.3390/ijms27156989 (PMC13467211; doi:10.3390/ijms27156989)
Supplement: Supplementary file 1 [file ijms-27-06989-s001.zip › Supplementary_Materials_last.pdf]

Supplementary Materials

A Generalizable and Interpretable Framework for Molecular Subtype Classification of Pancreatic Ductal Adenocarcinoma Integrating Conformal Uncertainty Quantification and Consensus-Based Explainable Artificial Intelligence Across Multiple Cohorts

Seyma Yasar, Firstname Lastname and Firstname Lastname

Figure S1

Figure S1. Tumor purity (neoplastic cellularity) distribution of the CPTAC-PDAC discovery cohort, derived from the histopathology-based Neoplastic_cellularity field of the CPTAC clinical table. The dashed line marks the 0.30 purity threshold; most CPTAC samples fall below it, consistent with the dense desmoplastic stroma of PDAC and the lower label-assignment margin observed in this cohort.

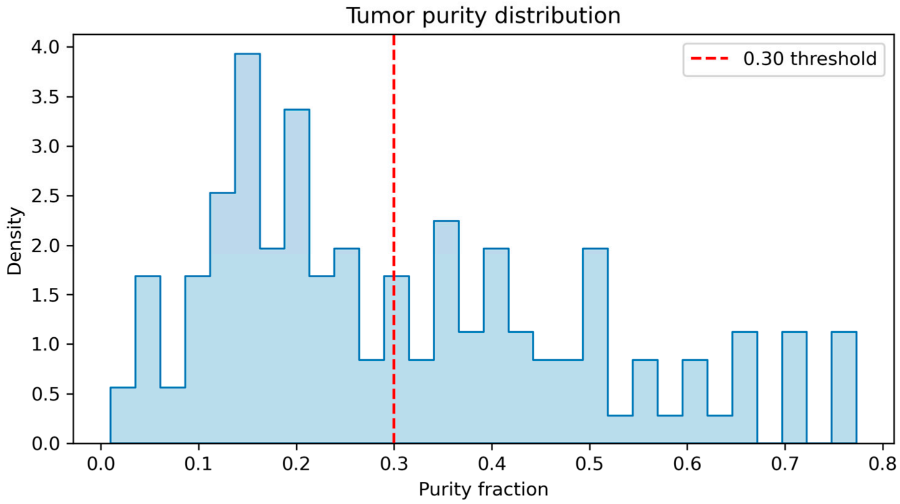

Figure S2

Figure S2. Conformal abstention rate as a function of the Moffitt label-assignment margin. Abstention concentrates on biologically borderline tumors (low margin) and falls to zero for high-margin, confidently subtyped samples.

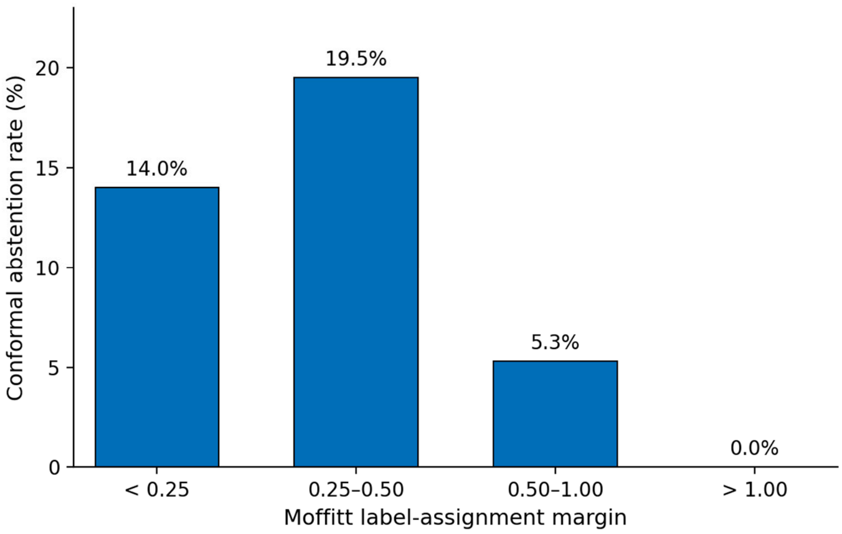

Table S1

Table S1. Histological composition of the TCGA-PAAD external cohort and the ductal-adenocarcinoma histology filter.

Histological type (TCGA-PAAD)	n (of 183)	Filter outcome
Pancreas — Adenocarcinoma, Ductal Type	150	Retained
Pancreas — Adenocarcinoma, other/unspecified subtype	27	Excluded
Pancreas — Colloid (mucinous non-cystic) carcinoma	4	Excluded
Pancreas — Undifferentiated carcinoma	1	Excluded
Histological discrepancy (unresolved)	1	Excluded
Total entering histology filter	183	—
Retained (ductal adenocarcinoma)	150	—
Excluded (non-ductal)	33	—

Only histologically confirmed pancreatic ductal adenocarcinoma was retained, because the Moffitt Classical/Basal-like signature is defined for PDAC; colloid (mucinous), undifferentiated and other-subtype adenocarcinomas, together with one sample carrying an unresolved histological discrepancy, were excluded. The retained cohort (n = 150) closely matches the approximately 150 bona-fide PDAC identified in the curated TCGA-PAAD analysis of Peran et al. (Clin Cancer Res 2018).

Table S2

Table S2. Four-method consensus explainable-AI (CXAI) ranking of the top 30 discriminative genes (data underlying Figure 4A). Of the 30 genes, 17 are canonical Moffitt-signature members and 13 are candidate subtype-informative genes.

Rank	Gene	SHAP	LIME	Permutation	DCA-FI	Mean CXAI
1	GPR87	0.98	0.98	0.837	0.98	0.944
2	A2ML1	0.918	0.939	0.918	0.898	0.918
3	FAM83A	0.898	1	0.878	0.878	0.913
4	PIP5K1B	0.816	0.816	0.98	1	0.903
5	GSDMC	1	0.959	0.633	0.959	0.888
6	IL20RB	0.959	0.857	0.408	0.939	0.791
7	TM4SF5	0.714	0.796	0.816	0.816	0.786
8	KRT16	0.694	0.735	0.939	0.755	0.781
9	AKR7L	0.878	0.837	0.653	0.694	0.765
10	LY6D	0.796	0.878	0.735	0.592	0.75
11	FSCN1	0.755	0.429	1	0.796	0.745

12	FMO5	0.857	0.918	0.571	0.388	0.684
13	CLDN18	0.837	0.898	0	0.918	0.663
14	PAQR8	0.939	0.776	0.041	0.837	0.648
15	FAM25A	0.653	0.673	0.265	0.857	0.612
16	IVL	0.735	0.755	0.898	0.061	0.612
17	RGS9BP	0.51	0.612	0.673	0.633	0.607
18	KIAA1211	0.612	0.531	0.959	0.163	0.566
19	IHH	0.531	0.633	0.531	0.469	0.541
20	ANKS4B	0.776	0.469	0.245	0.653	0.536
21	FOXA3	0.551	0.653	0.796	0.122	0.531
22	HNF4A	0.633	0.571	0.857	0	0.515
23	AKR7A3	0.408	0.327	0.714	0.571	0.505
24	GMDS	0.592	0.551	0.02	0.714	0.469
25	KRT9	0.122	0.347	0.776	0.612	0.464
26	LYZ	0.429	0.694	0.592	0.082	0.449
27	KLK5	0.49	0.49	0.082	0.735	0.449
28	TMEM40	0.367	0.449	0.184	0.776	0.444
29	KRT6A	0.327	0.592	0.449	0.286	0.413
30	GJB6	0.673	0.714	0.204	0.02	0.403

Table S3

Table S3. Overlap between the 50-gene consensus classification panel and the label-defining Moffitt 50-gene signature.

50-gene consensus classification panel	Count	Genes
Shared with Moffitt 50-gene signature	7 (14%)	CLDN18, FAM25A, FAM83A, GPR87, KRT6A, LY6D, LYZ
Outside the Moffitt signature	43 (86%)	—

Subtype labels were derived from the Moffitt 50-gene signature, raising a potential circularity concern. However, the externally validated 50-gene consensus classification panel shares only 7 genes (14%) with the Moffitt signature; 43 of 50 features (86%) lie outside it. Predictions also concord with the independent PurIST classifier (87.3% agreement on TCGA-PAAD). Together these indicate that the classifier captures a broader transcriptional programme correlated with, rather than identical to, the label-defining signature.

File S1

File S1. Completed TRIPOD+AI reporting checklist (27 of 30 items rated “Yes”, 2 “N/A”, 1 “Partial”), provided as a separate file.

Dataset S1

Dataset S1. Analysis-ready 50-gene consensus expression matrices and subtype labels for the CPTAC-PDAC (n = 140) and TCGA-PAAD (n = 150) cohorts, provided as the accompanying Excel workbook Supplementary_Dataset_S1_PDAC_subtype.xlsx.
